# Nature Notes: A new category for natural history studies

**DOI:** 10.1002/ece3.6534

**Published:** 2020-07-13

**Authors:** Allen J. Moore, Andrew P. Beckerman, Jennifer L. Firn, Christopher G. Foote, Gareth B. Jenkins

**Affiliations:** ^1^ Department of Entomology University of Georgia Athens GA USA; ^2^ Department of Animal & Plant Sciences University of Sheffield Sheffield UK; ^3^ School of Earth, Environmental & Biological Sciences Queensland University of Technology Brisbane QLD Australia; ^4^ John Wiley & Sons Ltd Oxford UK

*Ecology & Evolution* continues to be a journal that is author‐friendly and innovative. For example, we mandate data sharing, have no time for strict formatting guidelines or novelty as a barrier, and seek to introduce new manuscript categories. Our last such introduction, Academic Practice in Ecology & Evolution (Moore, Firn, & Beckerman, 2017) has proved to be a very popular option for authors over the last three years. Now, we are launching another novel avenue for authors—Nature Notes.

Natural history, a traditionally descriptive discipline, has been a key part of the development of the fields of ecology and evolutionary biology. However, the publication of such science has become more difficult over time as journals increasingly look to prioritize hypothesis‐driven research. Such studies should, and do, form the backbone of published work in our fields. However, that does not mean that descriptive and opportunistic observations should not have a place within the literature. We see this issue in reviewer reports as editors—reviews that find no fundamental issue with the science but feel it is too descriptive. We have actually always been happy to consider such work, but now we are formalizing that practice.

Nature Notes will be a category of article intended to remedy the issues that ecologists and evolutionary biologists can face in publishing “pure” natural history research and observations, which often do not adhere to the standard format of original research articles. We will evaluate Nature Notes submissions purely based on their contribution to our understanding of the natural world.

However, while we continue to apply our author‐friendly philosophy of novelty being a subjective criterion, we are not looking for purely confirmatory accounts. Instead, the emphasis will be placed on unusual or undescribed accounts of ecology and behavior. These could be species‐specific or community‐focussed, but we are looking beyond the routine and known. An analogy we like is to think of Nature Notes as the ecological equivalent of a clinical case report. For example, a report of a routine case is not worthy of publication. It needs to be some rare complication of a disease/unexpected reaction to a drug. Substitute in some relevant flora or fauna for drug, and you are there.

The first paper in this category in now online (Bhandari, Morley, Aryal, & Shrestha, 2020)— an article we hope will be the first of many. There is currently no set format, with the hope that the community will help us refine the full scope of how this article type moves forward. Interpret this as broadly as you want—we will work with you to get your work published. Short and succinct is preferable, but as we are online‐only you should feel free to structure your work as you see fit.

Nature Notes articles will be subject to the same standards of peer review and data accessibility as our other categories. This journal was founded to help authors have their work read and commented upon, regardless of perceived novelty or impact—we believe this will help with that goal.
